# Relationship between Running Spatiotemporal Kinematics and Muscle Performance in Well-Trained Youth Female Athletes. A Cross-Sectional Study

**DOI:** 10.3390/ijerph18168869

**Published:** 2021-08-23

**Authors:** Alejandro Castillo-Domínguez, Jerónimo C. García-Romero, Joaquín Páez-Moguer, Tomás Ponce-García, Miguel Medina-Alcántara, José Ramón Alvero-Cruz

**Affiliations:** 1 Department of Nursing and Podiatry, Ampliación Campus de Teatinos, University of Málaga, Arquitecto Francisco Peñalosa, 29071 Málaga, Spain; joaquinpaez@uma.es (J.P.-M.); migmedalc@uma.es (M.M.-A.); 2 Department of Human Physiology, Histology, Pathological Anatomy and Sports Physical Education, University of Málaga, 29071 Málaga, Spain; jeronimo@uma.es (J.C.G.-R.); tomas_ponce@uma.es (T.P.-G.); alvero@uma.es (J.R.A.-C.)

**Keywords:** muscle performance, biomechanics, kinematics, plyometrics, spatiotemporal, female, youth

## Abstract

The purpose of this cross-sectional study was to analyse the relationship of neuromuscular performance and spatiotemporal parameters in 18 adolescent distance athletes (age, 15.5 ± 1.1 years). Using the OptoGait system, the power, rhythm, reactive strength index, jump flying time, and jump height of the squat jump, countermovement jump, and eight maximal hoppings test (HT_8max_) and the contact time (CT), flying time (FT), step frequency, stride angle, and step length of running at different speeds were measured. Maturity offset was determined based on anthropometric variables. Analysis of variance (ANOVA) of repeated measurements showed a reduction in CT (*p* < 0.000) and an increase in step frequency, step length, and stride angle (*p* < 0.001), as the velocity increased. The HT_8max_ test showed significant correlations with very large effect sizes between neuromuscular performance variables (reactive strength index, power, jump flying time, jump height, and rhythm) and both step frequency and step length. Multiple linear regression found this relationship after adjusting spatiotemporal parameters with neuromuscular performance variables. Some variables of neuromuscular performance, mainly in reactive tests, were the predictors of spatiotemporal parameters (CT, FT, stride angle, and VO). Rhythm and jump flying time in the HT_8max_ test and power in the countermovement jump test are parameters that can predict variables associated with running biomechanics, such as VO, CT, FT, and stride angle.

## 1. Introduction

Distance running performance is mainly associated with physiological characteristics [1], such as running economy [2]. Traditionally, in adult athletes and also in adolescents [3,4], it is related to metabolic efficiency [5], the type of muscle fibres [6], and cardiorespiratory efficiency through heart rate [7] and VO_2max_ [8], which has also been previously associated with genetic variants [9,10]. However, there are other parameters, such as running biomechanics [11,12] and the ability to jump [13,14], which have also established a relationship with performance, suggesting that optimal movement performance and appropriate neuromuscular performance will also have a positive impact on energy cost parameters.

Among the spatiotemporal parameters most related to running economy are the contact time (CT), vertical oscillation (VO), step frequency and stride angle [12,15]. Some of these variables have also been associated with the prevalence of running-related injuries [16,17,18,19] together with growth-related factors, such as maturity offset and, more specifically in female athletes, the interrelationship of energy availability, menstrual function, and bone mineral density defined as the Female Athlete Triad [20,21]. The relationship between maturity offset and running biomechanics has been widely reported in sprint [22,23,24,25] and, more sparingly, in the distance running [26].

In short- and long-distance running, improved performance after training jumping ability has been associated with increased muscle strength and power [27], as well as stiffness of the muscular-tendinous system (which allows for the storage and use of elastic energy more efficiently) [5,28]. Previous studies have shown that combined strength and endurance training can increase running economy, muscle strength, and performance without affecting VO_2max_ [29], suggesting that endurance running performance may be affected by neuromuscular factors.

The assessment of the jump ability can be carried out using a variety of instruments, including accelerometric systems [30], force platforms, contact mats and optical systems [31], which can also be used for running biomechanical evaluation [32]. Countermovement, rebound [33], and multi-hopping jumps are used [34] to measure jump ability, which also evaluates the neuromuscular performance and efficiency of the stretch-shortening cycle in distance athletes [35]. Because the ability to develop strength in the shortest possible time is required in most sports [36,37,38], the reactive strength index has been developed as a reliable measure of force and time it takes to produce it [39]. Reliability and validity of its measurement in adolescents through the maximum hopping test has been proven [40] and has recently been recommended for development as part of the strength training of athletes, especially in women [41]. In addition, the increase in reactive strength index has been linked to performance improvements in middle-distance running [42].

Investigation of the relationship of spatiotemporal parameters with neuromuscular performance has shown different results [14,43,44,45,46], suggesting that neuromuscular factors could influence running biomechanics through kinematic spatiotemporal parameters. However, there are no studies that relate these biomechanical parameters to the neuromuscular performance of highly trained female adolescent athletes. This prevents drawing the same conclusions for different genders and sport levels. For this reason, the main objective of this study was to analyse the relationship between the running spatiotemporal parameters with neuromuscular performance (measured by the ability to jump [47]) in highly trained adolescent distance runners. The authors hypothesize that higher values of neuromuscular performance are related to lower VO, stride angle, and CT and an increase in FT.

## 2. Materials and Methods

This study was conducted according to the guidelines of the Declaration of Helsinki and approved by the Ethics Committee of the University of Málaga (CEUMA Registration number: 56-2019-H). Researchers obtained informed consent from all subjects involved in the study. Parents/guardians signed consent before participation, and in the case of those over 18 years of age, consent was provided by participants.

### 2.1. Participants

To conduct this cross-sectional study, 18 female adolescent athletes (age ± SD, 15.5 ± 1.1 years; age range, 14–18 years; height, 164.5 ± 8.2 cm; body mass, 55.5 ± 7.4 kg and body mass index, 20.5 ± 2.22 kg/m^2^) voluntarily participated in this study. Participants met the following inclusion criteria: age 14 to 18 years old, no injuries in the previous 3 months, and competed regionally, nationally, or internationally in medium-distance (800–3000 m) or long-distance (5–10 km and cross-country). Subjects were instructed to avoid training for 24 h before testing. All evaluations were carried out in a laboratory at 20–24 °C, with a relative humidity of 45–55% and similar conditions for all participants.

### 2.2. Procedure

Participants were summoned to perform the jumping and progressive running tests on the same day. First, primary anthropometric data were collected. Before starting the jumping and running tests, participants completed a warm-up phase with 10 min of continuous running and 5 min of activation exercises (knee lifts, accelerations, bracing, deep strides, and horizontal multi-hopping). The subjects then performed a battery of jumping tests: squat jump, countermovement jump, and eight maximal hoppings test (HT_8max_). The jumping tests were followed by a recovery period of 5 min. Subsequently, the participants conducted a running test on a motorized treadmill (Athlete 870C, Medisoft, Dinant, Belgium) in which spatiotemporal variables were measured. The grade of the slope was 1% when the spatiotemporal parameters were obtained [48]. Although footwear was not standardized among the participants, all used running shoes weighing less than 300 g. Participants had previous training experience on a treadmill [49]. They performed a standardized 10 min accommodation period divided into 5 min walking at 5 km·h^−1^ and 5 min running at 8 km·h^−1^, increasing the speed by 1 km·h^−1^ every 5 min until 12 km·h^−1^.

### 2.3. Materials and Assessment

#### 2.3.1. Anthropometric Assessment

For descriptive purposes, the height (cm) and body mass (kg) were determined through a stadiometer and a precision scale (Seca, Hamburg, Germany), and the body mass index of the participants was calculated based on body mass and height (kg/m^2^). All measurements were taken with participants wearing underwear. Anthropometric measurements were taken following the guidelines of the International Society for the Advancement of Kinanthropometry [50]. Each participant’s maturity offset was calculated using the formula described in [51]. This assessment is a non-invasive and practical method of predicting years from peak height velocity as a measure of maturity offset, using anthropometric variables.

#### 2.3.2. Spatiotemporal Assessment

Running spatiotemporal parameters were measured with the system previously validated for this purpose, OptoGait (Optogait, Microgate, Bolzano, Italy) [32,52]. The default settings for the filter 0_0 (Gait R. in: 0 and Gait R. out: 0 filter) were used. This configuration provides the least bias for time parameters in athletic walking [53]. Spatiotemporal parameters measured for each step during the 30 s uptake interval at 9 km·h^−1^, 10 km·h^−1^, and 11 km·h^−1^ were the contact time (CT, in seconds; time since the foot touches the ground until the toes separate from the ground), flight time (FT, in seconds; time from the take-off of the forefoot to the initial ground contact of the next contralateral support), vertical oscillation (VO, in centimetres; change in the height of the centre of gravity during the run), step frequency (in steps per minute; number of ground contacts per minute), step length (in metres; distance between two successive contacts with the ground, finger-to-finger) and stride angle (in degrees; the angle formed by the tangent of the parabola traced by the foot to the ground during a stride). The theoretical parabola for determining the stride angle was calculated by the system using the stride length and the maximum height of the foot during a stride [15].

#### 2.3.3. Neuromuscular Performance Assessment

Neuromuscular performance was measured by the jumping test using the same, previously validated [31], system (Optogait; Microgate, Bolzano, Italy). This device measures ground contact time and flight time using photoelectric cells. Flight time during the jump was used to calculate the jump height using the body’s centre of gravity. The participants carried out a familiarization session in which they could practice each of the jump protocols. The tests used in the study were in the following order: squat jump, countermovement jump, and HT_8max_. Two measurements were made for each jump test, and the best result obtained in each jump modality was chosen. Squat jump was performed starting from a 90° knee flexion position. Participants held this position for 2 s before jumping vertically to reach maximum height after an acoustic signal. In accordance with other studies, it was visually verified that no countermovement was performed during the squat jump [54]. To complete the countermovement jump, participants descended from an initial standing position to a sitting position, immediately followed by a vertical jump. Participants were encouraged to perform the eccentric phase of the jump as quickly as possible, with the depth of the countermovement phase selected by the participant to maximize jump height [33]. For squat jump and countermovement jump, power (using the formula proposed by Sayers et al. [55]), jump height, and jump flying time were obtained in each test. The percentage of elastic energy that contributed during the jump [56] was quantified by the elasticity index using the formula:

Elasticity Index = (countermovement jump_H_ − squat jump_H_) × 100/squat jump_H_ (1)

HT_8max_ consisted of performing eight repeated maximum vertical jumps. Participants were instructed to maximize jump height and minimize contact time with the ground during the jumps [34]. The first jump of each test served as a countermovement jump and was therefore discounted for analysis. The remaining seven jumps were averaged to analyse the jump contact time (s), jump flying time (s), jump height (cm), reactive strength index (m·s^−1^), rhythm (jumps·s^−1^), and power (w·kg^−1^) of each jump. Fatigue index, a variable that indicates the subject’s ability to maintain maximum force during the HT_8max_, was calculated as:

Fatigue index = (power_max_ − power_min_)/(power_max_ × 100) (2)

This percentage indicates the proportion of force the subject has maintained at the end of the continuous jumps, not the remaining power deficit. To be considered of maximum intensity, the mean of the jump height of the first three jumps needed to be higher than 95% of the jump height of the countermovement jump [47]. The reactive strength index was measured by the ratio between jump height and jump contact time (mm·ms^−1^) during HT_8max_ [39].

### 2.4. Statistical Analysis

Statistical analysis was performed using IBM-SPSS Statistics v. 25.0 (IBM Corp, Released 2017; Armonk, NY, USA). Data are presented as means and standard deviations. Normality was analysed using the Shapiro–Wilk test. An analysis of variance (ANOVA) of repeated measurements was conducted to study speed increase on spatiotemporal parameters. The association between variables was carried out using Pearson’s correlation coefficient.

An estimate of the effect size accompanied by the R^2^ scale was determined for Pearson’s correlations coefficient and ANOVA test. Effect sizes were classified as small, moderate, large, and very large (Table S1) [57,58].

A step-by-step multiple regression analysis was performed to determine the neuromuscular performance variables (non-dependent) predictors of spatiotemporal variables (dependent). In all these statistical tests, a significant value was considered when *p* < 0.05.

## 3. Results

Summary of participants’ characteristics and variables related to HT_8max_, squat, and countermovement jump are shown in Table 1. Regarding maturity offset values, all participants were considered post-pubertal (≥1.0 year), even when the SE associated with the prediction equation was taken into account [51]. A direct relationship was found between maturity offset and power of countermovement jump test (*r* = 0.523; *p* = 0.026) and squat jump test (*r* = 0.523; *p* = 0.026), with an effect size of 26.5% and 27.4%, respectively.

### 3.1. Running Spatiotemporal Variables

The ANOVA used to determine the effect of velocity on the spatiotemporal variables indicated that, as velocity increased, CT decreased significantly (*p* < 0.001) with a linear inverse relationship (0.89). FT, VO, step length, step frequency, and stride angle increased significantly (*p* < 0.001) as velocity increased, with a linear direct relationship (0.80, 0.88, 0.92, 0.83, and 0.69, respectively). A very large effect size (from 74.9% to 88.2%) was found between these variables (Table 2).

### 3.2. Linear Correlations between Spatiotemporal Variables and Jumping Tests

The correlation of the neuromuscular performance variables with the spatiotemporal parameters is shown in Table 3 for the HT_8max_ test and of the countermovement with squat jump tests in Table 4.

Regarding the HT_8max_ test, jump contact time presented moderate correlations (*r* > 0.30 in many cases and effect sizes > 9%) with CT, FT, VO, and stride angle, without finding a pattern which made us think that speed increased or decreased the correlations. In fact, the highest coefficients (*r* = 0.37; effect size of 13.9%) appeared at intermediate speed.

The jump flying time showed high direct coefficients with some spatiotemporal variables, such as step frequency at 11 km·h^−^^1^ (*r* = 0.68; effect size of 46.2%) and 9 km·h^−^^1^ (*r* = 0.58; effect size: 30%), and inverse with step length at 11 km·h^−^^1^ and at 9 km·h^−^^1^ (both also *r* = 0.67). Likewise, the coefficient was high (*r* = 0.50 to 0.59; effects size over 30%) and inverse with CT at different speeds.

The jump height had high correlations at different speeds with step length (*r* = −0.61 to −0.64; about 40% effect size), step frequency (*r* = 0.57 to 0.65), and CT (*r* = 0.48 to 0.56; effect size about 27%). These results were similar to variable rhythm, but in different directions. In this case, the correlation was high with step length (*r* = 0.61 to 0.63; effect size over 38%), step frequency (*r* = −0.56 to −0.64), and CT (*r* = 0.57 to 0.63). In this case, there was also a moderate correlation (*r* = 0.31; effect size of 9.6%) with FT at 9 km·h^−1^ and VO at 9 km·h^−1^ (*r* = 0.24; effect size of 5.8%).

Reactive strength index showed high correlation coefficients with step length (between −0.58 and −0.65) and step frequency (between 0.54 and 0.64) with very large effect sizes (34.1% to 42.6%). A somewhat lower intensity coefficient was found for CT (between 0.37 and 0.49) in the inverse direction and large effect sizes (13.9% to 24%). There were similar results for power, resulting in high correlations and very large effect size with step length (*r* = −0.6 to −0.66; effect size about 40%) and step frequency (*r* = 0.552 to 0.659; effect size about 36.5%).

Regarding the fatigue index, we found moderate correlations (*r* = 0.31 to 0.45), accompanied by moderately large effect sizes (effect sizes were >10% in many cases), with FT, VO, step length, step frequency, and stride angle.

In an overall assessment of the squat and countermovement jump, these results indicated slight relationships between these tests and spatiotemporal variables (Table 4). A more detailed analysis showed that jump flying time and jump height in both tests correlated moderately (between 0.27 and 0.32; 9% effect size) with step frequency at different speeds. This relationship was direct, thus associating high values of countermovement jump variables with high step frequency values.

Power of countermovement jump test showed higher correlations with CT, FT, VO, and stride angle (*r* = 0.3 to 0.5; effect sizes 9 to 25%), direct with CT and inverse with FT, VO, and stride angle. Similar results were obtained for elasticity index, showing moderate correlations with CT, FT, VO, and stride angle (*r* > 0.4 in many cases; effect size about 20%), direct with CT and inverse with FT, VO, and stride angle.

The relationships found between the pairs of variables reactive strength index, jump flying time, jump height, rhythm, and power with step length, step frequency, and CT ceased to be significant after adjustment for the variables of neuromuscular performance (jump contact time, jump flying time, jump height, rhythm, power, reactive strength, fatigue, and elasticity indexes) in the multivariate study (Table 5).

The two-step stride angle prediction model (adjusted R^2^ = 0.524) initially included the jump flying time of HT_8max_ (R^2^ = 0.439, *p* = 0.003) and, later, the power of countermovement jump (R^2^ = 0.58, *p* = 0.04), explaining 52% of the variance for stride angle.

For the HT_8max_, the rhythm was a negative predictor of 22% of the CT (B = −0.043, *p* = 0.027) and a positive predictor of 34% of the FT variance (B = 0.054, *p* = 0.006), and the jump flying time was a negative predictor (B = −3.983, *p* = 0.002) of 42% of the variance for VO.

## 4. Discussion

To our knowledge, this study is the only one that analysed the relationship of running spatiotemporal parameters with neuromuscular performance in well-trained adolescent runners. It was anticipated that the neuromuscular performance might have a relationship with spatiotemporal variables, such as CT, FT, VO, or stride angle, resulting in optimizing the effective energy transfer during contact with the ground at different speeds [35,39]. In this study, neuromuscular performance variables related to running spatiotemporal parameters have been identified. Specifically, a higher number of jumps per second and average flight time during the HT_8max_ multi-jump test were predictors of shorter contact times, vertical oscillations and step angle, as well as longer flight times during running.

Regarding adaptations of spatiotemporal parameters after the increase in velocity, the results of this study reinforced the findings of previous studies [43,59,60]. Increased running speed has been shown to lead to a reduction in CT (to facilitate the progression of the leg during the oscillation phase to a new contact [59]) and to an increase in step frequency, as a spatiotemporal adaptation needed to run faster [61]. In addition, CT has been linked as a determinant of leg stiffness, with higher values of leg stiffness associated with shorter CT [61]. The increase in stride angle was also observed at a higher rate, agreeing with those obtained in previous studies [43,60].

Although the influence of neuromuscular factors on performance in endurance athletes seems clear [5,28,29,35,42], current findings suggest that combined training of neuromuscular performance and endurance does not produce changes in step length and frequency during running at the constant submaximal speed [62]. Roche-Seruendo et al. [43] also measured the neuromuscular performance of amateur adult runners by jumping capacity, finding a lack of influence of the neuromuscular performance on spatiotemporal adaptations produced during the increase in running speed. Gómez-Molina et al. [44] found an increase in FT and step length (decreasing the step frequency and keeping the CT constant) in those runners who did the run and jump training. In contrast, isolated running training slightly reduced FT and step length (increasing step frequency), as previously suggested by other authors, but without analysing these variables [13]. These studies only included amateur male participants, so they did not evaluate gender differences or the relationship of these variables in professional athletes, which seem to associate increased step frequency with a lower risk of injury and higher running economy than novices [63].

This study showed that the rhythm of multi-hopping (jump per second) can predict CT and FT at 22% and 34% of the total variance, respectively, indicating that a higher rhythm could induce lower CT and higher FT. These findings coincide with the study by Paavolainen et al. [29], which showed that training of neuromuscular performance through reactive force exercises in well-trained adult athletes significantly decreased CT during running, without observing changes in step length or frequency, and assuming an increase in FT. However, Ferrauti et al. [45] observed that neuromuscular performance training through maximum strength exercises increased CT during running. These results reinforce the idea that reactive strength exercises (such as HT_8max_) emphasize the development of strength in lower CTs [62], suggesting that by testing this skill during training, athletes could adjust the CT with the ground (transferring this adaptation to running). Furthermore, jumping ability training in adolescent females produces a more significant improvement in reactive tests related to multi-jumping than countermovement and squat jump [64], with reactive strength exercise training effects revealing a trivial effect (ES = 0.19) on jumping performance in elite female runners [65]. This could explain the existence of stronger relationships between the variables of the HT_8max_ test and running kinematics compared with the squat and countermovement jump tests. However, analysis of maturity offset on the neuromuscular performance variables revealed that more mature athletes might be able to produce a greater amount of power in the countermovement and squat jump tests with significant relationship (*r* = 0.523; *p* = 0.026) and very large effect size of 26.5% and 27.4%, respectively. Similar results in the countermovement and squat jump tests have recently been reported by Dobbs et al. [66], with very large increases in neuromuscular performance with increasing maturity status.

Modifications in spatiotemporal parameters, such as reduced CT including increased step frequency to 180 steps per minute, have recently been associated with 8.7% lower oxygen consumption [67] in well-trained adult female athletes and decreased risk of running-related injuries [16,17,18,19]. The increase in step frequency is one of the most used running retraining strategies from a clinical point of view because its increase above the preferred cadence at a constant speed generates a proportional shortening of the step length [68]. Despite increasing the number of loading cycles over a given distance, this will result in a reduction of the maximum moment of force and adduction of the hip, the reactive forces of the ground and tibial acceleration, the joint demand of the leg, and the velocity and vertical oscillation of the centre of mass [16,69]. The reduction of vertical displacement during the run may induce a lower metabolic cost associated with lower vertical impulses that seek to maintain body weight concerning severity [12]. This study found that jump flying time during HT_8max_ would explain 42.5% of the total variance in VO magnitude in well-trained adolescent runners.

The results obtained would indicate that greater development of force in shorter times [35,39] by improving the rhythm or jump flying time of the HT_8max_ could be associated with an increase in FT (reducing the CT and VO) during running. These optimizations could improve the ability to achieve better energy transfer during contact with the ground by increasing the FT and minimizing the CT efficiently, thus avoiding wasted vertical movements. In addition, the model based on the jump flying time in the HT_8max_ test and the power in the countermovement jump test would explain 52.4% of the total variance of stride angle, with power obtained in the countermovement jump test being a significant positive predictor of stride angle. Higher values of stride angle have been associated with the improvement of running efficiency through the early contraction of the muscles involved in shifting the centre of mass during the stride in adult elite runners [15].

### Limitations

Despite the homogeneity of the sample, the small number of subjects who participated in this study had limitations when it came to generalizing results and could explain that the relationship between running spatiotemporal parameters and the fatigue index with moderate or large effects did not reach significance (*p* > 0.05). In addition, the depth jump or countermovement jump tests could also have been used for the measurement of reactive strength index, instead of the HT_8max_ repeated jump test. However, the proven reliability and validity of the reactive strength index measurement through the multi-hopping test in adolescents [40] determined its choice as a measurement method. Interpretations in the general population should therefore be made with caution. Future studies in the general population that would relate variables of the neuromuscular performance (such as reactive strength index, power, or jump height) with running kinematics and other sporting gestures that involve the stretch-shortening cycle, are needed. The relationship of these variables with parameters that could play an essential role during running, such as running economy and reactive forces of the ground (running kinetics), could be studied. The study of the relationship between neuromuscular performance and biomechanical parameters in speed athletes could improve our understanding of the relationship between these variables at higher speeds. During training for long-distance running, an increase in rhythm and jump flying time could improve performance and reduce injuries due to a reduction in CT and VO. This would have significant implications to consider when coaches or athletes plan running technique exercises during training sessions. Rhythm, jump flying time, and power are easy to obtain and valid measurements that can be useful for any athletics coach, considering that their acquisition by handheld devices is feasible.

## 5. Conclusions

This study described the predictive capacity of muscular performance concerning running spatiotemporal parameters in well-trained adolescent women.

The HT_8max_ test had a higher predictive power for the countermovement and squat jump, indicating that reactive force tests have a higher predictive capacity of running spatiotemporal parameters than the maximum or explosive strength tests.

Training variables associated with reactive jump tests (such as the higher rhythm of jumps and average flight time) were associated with shorter contact times, longer flight times, and lower vertical oscillations during running.

The explosive jump test was a better predictor of stride angle. An increase in power during this test was associated with higher stride angle values during running.

## Figures and Tables

**Table 1:** Demographic and jumping test characteristics of the participants.

Variables	Mean ± SD
Height (cm)	164.5 ± 8.2
Body mass (kg)	55.5 ± 7.4
Body mass index (kg/m^2^)	20.5 ± 2.22
Maturity offset (years)	3.1 ± 1
Squat Jump	
Jump Flying Time (s)	0.45 ± 0.04
Jump Height (cm)	25.54 ± 4.36
Power (W)	2008.93 ± 400.31
Countermovement Jump	
Jump Flying Time (s)	0.47 ± 0.04
Jump Height (cm)	26.99 ± 4.32
Power (W)	2107.50 ± 391.82
8 Maximal Hopping Test (HT_8max_)	
Jump Flying Time (s)	0.42 ± 0.05
Jump Height (cm)	22.25 ± 5.04
Jump contact time (s)	0.2 ± 0.02
Rhythm (jumps·s^−1^)	1.63 ± 0.16
Reactive strength index (m·s^−1^)	1.15 ± 0.26
Power average (W·Kg^−1^)	32.68 ± 6.11
Power min (W·Kg^−1^)	28.59 ± 5.55
Power max (W·Kg^−1^)	36.07 ± 6.38
Fatigue Index (%)	20.82 ± 5.77
Elasticity Index (%)	6.07 ± 5.42

**Table 2:** Repeated measurements ANOVA. Effect of velocity on spatiotemporal variables.

Variables	Velocity			ANOVA			Linear Adjust
	9 km·h^−1^	10 km·h^−1^	11 km·h^−1^	*f* Value	*p* Value	R^2^	
CT (s)	0.28	0.26	0.24	85.27 **	<0.0001	0.834	0.89
FT (s)	0.10	0.11	0.12	50.63 **	<0.0001	0.749	0.80
VO (cm)	1.21	1.44	1.79	72.53 **	<0.0001	0.810	0.88
Step length (cm)	94.84	101.96	110.10	127.07 **	<0.0001	0.882	0.92
Step frequency (steps/min)	160.98	163.61	166.35	56.01 **	<0.0001	0.767	0.83
Stride angle (°)	2.89	3.23	3.73	24.22 **	<0.0001	0.588	0.69

** = highly significant; CT = contact time; FT = flying time; VO = vertical oscillation.

**Table 3:** Correlation coefficients and estimation of effect size between spatiotemporal variables and HT_8max_ at 9 km·h^−1^, 10 km·h^−1^, and 11 km·h^−1^.

		HT_8max_						
		Jump Contact Time (s)	Jump Flying Time (s)	Jump Height (cm)	Rhythm (Jumps/s)	Reactive Strength Index (m/s)	Power (W/Kg)	Fatigue Index (%)
9 km·h^−1^								
CT (s)	*r*	−0.307	−0.509 *	−0.492 *	0.573 *	−0.402	−0.426	−0.142
	R^2^	0.094	0.259	0.242	0.328	0.162	0.181	0.02
FT (s)	*r*	0.317	0.223	0.217	−0.312	0.133	0.149	0.403
	R^2^	0.101	0.05	0.047	0.098	0.018	0.022	0.162
VO (cm)	*r*	0.279	0.160	0.156	−0.23	0.082	0.095	0.402
	R^2^	0.078	0.025	0.024	0.057	0.007	0.009	0.161
Step length (cm)	*r*	−0.041	−0.675 **	−0.641 **	0.616 **	−0.653 **	−0.666 **	0.424
	R^2^	0.002	0.455	0.411	0.379	0.426	0.443	0.18
Step frequency (steps/min)	*r*	0.068	0.586 *	0.568 *	−0.556 *	0.535 *	0.552 *	−0.389
	R^2^	0.005	0.343	0.322	0.309	0.286	0.305	0.152
Stride angle (°)	*r*	0.267	0.300	0.289	−0.366	0.221	0.237	0.317
	R^2^	0.071	0.09	0.084	0.134	0.049	0.056	0.1
10 km·h^−1^								
CT (s)	*r*	−0.340	−0.497 *	−0.479 *	0.570 *	−0.373	−0.401	−0.078
	R^2^	0.115	0.247	0.230	0.325	0.139	0.160	0.006
FT (s)	*r*	0.373	0.112	0.110	−0.227	0.001	0.020	0.367
	R^2^	0.139	0.013	0.012	0.051	0	0	0.135
VO (cm)	*r*	0.364	0.067	0.064	−0.181	−0.046	−0.026	0.376
	R^2^	0.133	0.004	0.004	0.033	0.002	0.001	0.142
Step length (cm)	*r*	−0.065	−0.639 **	−0.612 **	0.611 **	−0.584 *	−0.602 **	0.314
	R^2^	0.004	0.408	0.375	0.373	0.341	0.363	0.099
Step frequency (steps/min)	*r*	0.094	0.646 **	0.621 **	−0.624 **	0.581 *	0.601 **	−0.334
	R^2^	0.009	0.417	0.385	0.389	0.337	0.361	0.111
Stride angle (°)	*r*	0.369	0.148	0.142	−0.257	0.031	0.052	0.338
	R^2^	0.136	0.022	0.02	0.066	0.001	0.003	0.114
11 km·h^−1^								
CT (s)	*r*	−0.240	−0.588 *	−0.561 *	0.630 **	−0.490 *	−0.514 *	0.050
	R^2^	0.058	0.346	0.314	0.397	0.24	0.264	0.002
FT (s)	*r*	0.329	−0.073	−0.073	−0.045	−0.167	−0.160	0.448
	R^2^	0.109	0.005	0.005	0.002	0.031	0.026	0.201
VO (cm)	*r*	0.305	−0.133	−0.129	0.024	−0.229	−0.215	0.419
	R^2^	0.093	0.018	0.017	0.001	0.052	0.046	0.175
Step length (cm)	*r*	−0.008	−0.671 **	−0.641 **	0.633 **	−0.642 **	−0.656 **	0.388
	R^2^	0	0.451	0.411	0.401	0.412	0.43	0.151
Step frequency (steps/min)	*r*	0.024	0.678 **	0.650 **	−0.639 **	0.643 **	0.659 **	−0.375
	R^2^	0.001	0.46	0.423	0.408	0.414	0.434	0.141
Stride angle (°)	*r*	0.325	0.030	0.026	−0.135	−0.079	−0.061	0.350
	R^2^	0.105	0.001	0.001	0.018	0.006	0.004	0.122

CT = contact time; FT = flying time; VO = vertical oscillation; HT_8max_ = eight maximal hoppings test; R^2^ = coefficient of determination R square; *r* = correlation coefficient. * *p* < 0.05, ** *p* < 0.01.

**Table 4:** Correlation coefficients and estimation of effect size between spatiotemporal biomechanical variables and countermovement—squat jump at 9 km·h^−1^, 10 km·h^−1^, and 11 km·h^−1^.

		Countermovement Jump			Squat Jump			Elasticity Index (%)
		Jump Flying Time (s)	Jump Height (cm)	Power (W/Kg)	Jump Flying Time (s)	Jump Height (cm)	Power (W/Kg)	
9 km·h^−1^								
CT (s)	r	0.057	0.066	0.429	−0.060	−0.057	0.317	0.347
	R^2^	0.003	0.004	0.184	0.004	0.003	0.1	0.134
FT (s)	r	−0.196	−0.213	−0.406	−0.040	−0.052	−0.292	−0.427
	R^2^	0.038	0.045	0.165	0.002	0.003	0.085	0.208
VO (cm)	r	−0.194	−0.212	−0.457	−0.044	−0.057	−0.341	−0.424
	R^2^	0.038	0.045	0.209	0.002	0.003	0.117	0.19
Step length (cm)	r	−0.182	−0.193	0.239	−0.127	−0.143	0.223	−0.067
	R^2^	0.033	0.037	0.057	0.016	0.021	0.05	0.026
Step frequency (steps/min)	r	0.316	0.318	−0.136	0.299	0.305	−0.088	−0.160
	R^2^	0.1	0.101	0.018	0.089	0.093	0.008	0.002
Stride angle (°)	r	−0.156	−0.172	−0.500 *	−0.013	−0.023	−0.378	−0.284
	R^2^	0.024	0.03	0.25	0	0.001	0.143	0.177
10 km·h^−1^								
CT (s)	r	−0.113	−0.097	0.426	−0.240	−0.231	0.284	0.479 *
	R^2^	0.013	0.009	0.182	0.058	0.053	0.081	0.209
FT (s)	r	−0.079	−0.099	−0.306	0.097	0.081	−0.172	−0.568 *
	R^2^	0.006	0.01	0.094	0.009	0.007	0.03	0.319
VO (cm)	r	−0.055	−0.078	−0.351	0.117	0.099	−0.212	−0.542 *
	R^2^	0.003	0.006	0.123	0.014	0.01	0.045	0.309
Step length (cm)	r	−0.253	−0.252	0.309	−0.237	−0.241	0.252	0.155
	R^2^	0.064	0.063	0.095	0.056	0.058	0.064	0
Step frequency (steps/min)	r	0.280	0.279	−0.325	0.269	0.274	−0.259	−0.191
	R^2^	0.078	0.078	0.106	0.072	0.075	0.067	0.002
Stride angle (°)	r	−0.035	−0.057	−0.381	0.137	0.120	−0.236	−0.545 *
	R^2^	0.001	0.003	0.145	0.019	0.014	0.056	0.316
11 km·h^−1^								
CT (s)	r	−0.106	−0.097	0.433	−0.211	−0.212	0.303	0.447
	R^2^	0.011	0.009	0.188	0.045	0.045	0.092	0.13
FT (s)	r	−0.269	−0.283	−0.359	−0.106	−0.115	−0.253	−0.401
	R^2^	0.072	0.08	0.129	0.011	0.013	0.064	0.193
VO (cm)	r	−0.211	−0.230	−0.389	0.054	−0.070	−0.278	−0.437
	R^2^	0.044	0.053	0.151	0.003	0.005	0.077	0.197
Step length (cm)	r	−0.229	−0.238	0.187	−0.204	−0.219	0.149	0.145
	R^2^	0.052	0.057	0.035	0.042	0.048	0.022	0.001
Step frequency (steps/min)	r	0.283	0.287	−0.210	0.267	0.278	−0.157	−0.155
	R^2^	0.08	0.082	0.044	0.071	0.077	0.025	0.001
Stride angle (°)	r	−0.168	−0.187	−0.453	−0.008	−0.021	−0.326	−0.445
	R^2^	0.028	0.035	0.205	0	0	0.107	0.216

CT = contact time; FT = flying time; VO = vertical oscillation; R^2^ = coefficient of determination R squared; *r* = correlation coefficient. * *p* < 0.05.

**Table 5:** Multiple linear regression model testing the significative association between spatiotemporal variables with muscular performance.

	Rhythm (HT_8max_)			Jump Flying Time (HT_8max_)			Power (Countermovement Jump)		
Dependent Variables	R^2^	B (SE)	*p*	R^2^	B(SE)	*p*	R^2^	B(SE)	*p*
CT (s)	0.27	−0.043 (0.018)	0.027						
adjusted R^2^		0.224							
FT (s)	0.38	0.054 (0.017)	0.006						
adjusted R^2^		0.341							
VO (cm)				0.458	−3.983 (1.082)	0.002			
adjusted R^2^					0.425				
Stride angle (°) Model 1				0.439	−9.113 (2.577)	0.003			
adjusted R^2^					0.404				
Stride angle (°) Model 2				0.58	−9.324 (2.305)	0.001	0.58	0.001 (0)	0.04
adjusted R^2^					0.524			0.524	

R^2^ = coefficient of determination R square; B = coefficient; SE = standard error; CT = contact time; FT = flying time; VO = vertical oscillation. Model 1: adjusted for jump flying time (HT_8max_). Model 2: adjusted for jump flying time (HT_8max_) and power (countermovement jump).

## Data Availability

The datasets used and/or analyzed during the current study are available from the corresponding author on reasonable request.

## Supplementary Materials

The following are available online at https://www.mdpi.com/article/10.3390/ijerph18168869/s1, Table S1. Categorization of effect size [58].
