# Supplementary material for: Relationship between Running Spatiotemporal Kinematics and Muscle Performance in Well-Trained Youth Female Athletes. A Cross-Sectional Study
Source: Int J Environ Res Public Health. 2021 Aug 23;18(16):8869. doi: 10.3390/ijerph18168869 (PMC8392471; doi:10.3390/ijerph18168869)
Supplement: Supplementary file 1 [file ijerph-18-08869-s001.zip › ijerph-1304054-supplementary.pdf]

Table S1. Categorization of effect size [58].

Small		Moderate		Large		Very large	
R^2	r	R^2	r	R^2	r	R^2	r
<	<	0.040	0.200	0.125	0.354	0.265	0.514
0.002	0.050	0.059	0.243	0.168	0.410	0.267	0.545
0.010	0.100	0.083	0.287	0.200	0.447	0.329	0.573
0.022	0.148	0.096	0.309	0.232	0.482	0.360	0.600
0.030	0.172	0.109	0.330	0.250	0.500	>	>

R^2 = coefficient of determination R square; r = correlation coefficient.
